# The influence of short-term heat acclimation with permissive dehydration on temperate exercise performance in highly trained athletes

**DOI:** 10.1186/2046-7648-4-S1-A109

**Published:** 2015-09-14

**Authors:** Rebecca Neal, Jo Corbett, Heather Massey, Michael J Tipton

**Affiliations:** 1 Extreme Environments Laboratory, Department of Sport and Exercise Science, University of Portsmouth, Portsmouth, UK

## Introduction

Long-term (≥ 10 day) heat acclimation (HA) has been shown to be ergogenic under cool ambient conditions [1]. Potential mechanisms underpinning the ergogenic effects of long-term HA include increased maximal oxygen uptake, possibly mediated by plasma volume (PV) expansion and an increased maximal cardiac output [1], as well as reduced physiological strain through improved thermoregulation [2]. Recently, short-term (5 day) HA with restricted fluid intake (STHADe) has been shown to augment PV expansion and accelerate HA relative to euhydrated HA [3]; performance improvements in the heat have been documented in highly-trained men following this regime [4]. This study examined the ergogenic effect of STHADe on exercise in a temperate environment.

## Methods

Ten highly-trained male cyclists and triathletes (Mean[SD] age 24[4] years; height 1.76[0.04] m; mass 70.9[7.3] kg; maximal oxygen uptake [VO_2max_]: 63.3[4.0] mL.kg^-1^.min^-1^; peak power output [PPO]: 385[40] W; training: 10[3] hours.week^-1^) underwent a STHADe programme consisting of 5-consecutive days of exercise (90 mins.day^-1^) under isothermic heat strain (target rectal temperature [*T_re_*] of 38.5-38.9 °C) in a hot environment (*T_amb _*= 40 °C, 50 % rh). During HA sessions, and for 30 minutes after, participants did not receive any fluids. Euhydrated heat stress tests (HST) were completed the day before and the day after the STHADe (60 mins cycling at 35 % PPO, *T_amb_*= 40 °C, 50 % rh). A graded exercise test (GXT) for determination of blood lactate threshold (LT), VO_2max _and PPO as well as a 20 km self-paced time trial (TT) (on a separate day) were performed in a temperate environment (22 °C, 50 % rh) pre- and post-STHADe.

## Results

STHADe significantly reduced rectal (Δ*T_re _*= -0.2[0.2] °C) and mean body temperature (Δ*T*_b _= -0.2[0.2] °C), heart rate (Δ*f*_c_= -7[7] b.min^-1^) and perceived exertion, and augmented local and whole body sweat rate (all *P*<0.05) during the HST; no clear expansion of plasma volume was seen (ΔPV = 1.2[8.0] %, *P *= 0.64). Constant workload exercise in a temperate environment indicated that STHADe reduced resting and exercising mean skin temperature (*T*_sk_), *T_re_, T*_b _and *f*_c _(all *P*<0.05) under these conditions. Performance trials in a temperate environment suggest that PPO (Δ= 6[7] W) and LT (Δ = 16[17] W) in the GXT were improved (*P *< 0.05) following STHADe but VO_2max _and TT performance were not significantly affected (*P *> 0.05) although there was a trend for a higher mean power (*P *= 0.06).

## Discussion

These data show typical markers of HA during exercise in the heat and that STHADe is effective at reducing thermal and cardiovascular strain under temperate conditions. The lack of ΔPV may be due to high baseline blood volumes in this highly trained cohort, or higher daily dehydration levels than in previous studies [3]. Although there was no effect on TT performance, other indicators of performance such as PPO were improved. These ergogenic effects might occur by thermal effects, such as a reduced 'physiological cost' of thermoregulation, or non-thermal effects, such as an improved power at LT.

## Conclusion

STHADe induced favourable thermal, thermoregulatory, physiological and cardiovascular responses to exercise in hot and temperate environments in highly-trained athletes. It may be necessary to extend the duration of HA to fully elucidate the ergogenic benefit in temperate environments.
